# Sertraline treatment influences [^18^F]FE-PE2I PET imaging for Parkinsonism

**DOI:** 10.1186/s13550-023-01000-6

**Published:** 2023-05-23

**Authors:** Thomas E. H. Justesen, Per Borghammer, Joel Aanerud, Peter Hovind, Lisbeth Marner

**Affiliations:** 1 Department of Clinical Physiology and Nuclear Medicine, Copenhagen University Hospital Bispebjerg, Bispebjerg Bakke 23, Copenhagen, Denmark; 2 Department of Nuclear Medicine and PET, Aarhus University Hospital, Aarhus N, Denmark; 3 Department of Clinical Medicine, University of Copenhagen, Copenhagen, Denmark

**Keywords:** Dopamine transporter, SSRI, Blocking, DAT, Positron emission tomography, Parkinson’s disease

## Abstract

**Background:**

The dopamine transporter (DaT) PET ligand [^18^F]FE-PE2I is used to aid the diagnosis of Parkinson’s disease. After encountering four patients with a history of daily sertraline use, who all showed atypical findings on [^18^F]FE-PE2I PET, we suspected that the selective serotonin reuptake inhibitor (SSRI), sertraline, might interfere with the results and lead to globally reduced striatal [^18^F]FE-PE2I binding due to sertraline’s high affinity for DaT.

**Methods:**

We rescanned the four patients with [^18^F]FE-PE2I PET after a 5-day sertraline pause. Sertraline plasma concentration was estimated based on body weight and dose, and specific binding ratios (SBR) in caudate nucleus, known to be more preserved in Parkinson’s, were used to estimate the effect on tracer binding. Comparison was made to a patient with [^18^F]FE-PE2I PET before and after a 7-day Modafinil pause.

**Results:**

We found a significant effect of sertraline on caudate nucleus SBR (*p* = 0.029). The effect showed a linear dose-dependent relationship that corresponds to a reduction in SBR by 0.32 or 0.44 for a 75 kg male or a 65 kg female, respectively, taking a daily dose of 50 mg sertraline.

**Conclusion:**

Sertraline is one of the most commonly used antidepressants and in contrast to other SSRI’s, sertraline show high affinity for DaT. We recommend that sertraline treatment is taken into account when patients are undergoing [^18^F]FE-PE2I PET especially in patients showing apparent globally reduced PE2I binding. If tolerable, pausing of the sertraline treatment should be considered, especially for doses above 50 mg/day.

**Supplementary Information:**

The online version contains supplementary material available at 10.1186/s13550-023-01000-6.

## Introduction

Dopamine transporter (DaT) imaging is commonly used for diagnosing Parkinson’s disease or atypical Parkinsonism. DaT imaging allows assessment of the presynaptic dopaminergic function in striatum through visualization of the DaT, which is found primarily in presynaptic nigrostriatal dopaminergic projections. In Parkinson’s disease, these dopaminergic synapses are lost along with the dopaminergic neuron cell bodies due to the accumulation of misfolded alpha-synuclein [1]. Several imaging centres have recently implemented and validated [^18^F]FE-PE2I PET to replace [^123^I]FP-CIT SPECT for DaT imaging to achieve higher resolution and shorter examination times [2–5]. An additional advantage of [^18^F]FE-PE2I PET is the selective binding to the DaT [6], while [^123^I]FP-CIT is known to also bind to serotonin transporters [7, 8].

Before DaT-imaging, patients should pause any medications that could occupy the DaT and potentially lead to false positive findings, including drugs like Modafinil (for narcolepsy), methylphenidate (for ADHD), recreational drugs (cocaine, amphetamine) and to a lesser extent the antidepressants mazindol, bupropion and radafaxine [1]. However, sertraline might also interfere with DaT-imaging. Sertraline is a selective serotonin reuptake inhibitor (SSRI) commonly used as first line treatment in mild to severe depression. Although targeting the serotonin transporter, sertraline possesses an equilibrium dissociation constant (*K*_D_) of 25 ± 2 nM for the DaT, and thus a high affinity for the DaT in contrast to other SSRI’s [9]. Thus, sertraline may potentially block the DaT interfering with [^18^F]FE-PE2I binding. As sertraline is widely used, also in elderly patients [10], the high DaT-affinity of sertraline could be a clinically significant problem.

We present data on four patients on sertraline treatment referred for [^18^F]FE-PE2I imaging under suspicion of Parkinson’s disease. Due to apparent globally reduced striatal [^18^F]FE-PE2I binding and suspicion of DaT blocking, the patients were rescanned after pausing sertraline. The results are compared to imaging of a patient treated with 100-mg Modafinil daily—a known DaT blocker.

## Methods

### Subjects

Four patients referred to DaT-imaging due to clinical suspicion of Parkinson’s disease showed globally reduced [^18^F]FE-PE2I uptake despite no record of known DaT blocking medication. Carefully re-examining the prescriptions again, we noticed that all patients took sertraline on a daily basis. Patients were offered repeated DaT-imaging preceded by a 5-day pause, since sertraline has a plasma half-life of ~ 24–26 h [11]. For comparison, a patient on Modafinil treatment was included. All patients gave written consent for publication. To avoid discontinuation syndrome, the daily sertraline treatment for patient 3 was switched to another SSRI during the sertraline pause. No discontinuation symptoms were observed. Please refer to Table 1 for demographic and clinical data.

**Table 1:** Demographic and clinical data

	Patient 1	Patient 2	Patient 3	Patient 4	Modafinil patient
Gender	Female	Female	Female	Male	Male
Age	58	74	78	76	65
Daily sertraline intake (mg)	100	100	125	50	100 (Modafinil)
Time of daily sertraline intake	Evening	Evening	Morning	Evening	–
Symptoms suggesting Parkinsonism	Action tremor, rigidity and dysdiadochokinesia	Initially treated for essential benign tremor, but no effect of treatment, and now action tremor as well	Fall, gait disturbances, hallucinations and cognitive decline	Cognitive decline, gait disturbances, apathetic, fall and urine incontinence	Unilateral tremor, slow movements, obstipation, hyposmia and reduced balance
CT/MRI findings	Sequelae in relation to previous infarction in the right hemisphere	Leukoaraiosis (Fazekas gr I/II)	Enlarged ventricular system and leukoaraiosis (Fazekas gr III)	Pronounced atrophy, leukoaraiosis and sequelae in relation to previous lacunar infarction	Light frontal atrophy and leukoaraiosis (Fazekas gr I/II)
Comorbidities	Diabetes type II, depression, hypertension and hypothyroidism	Previous gastric cancer (radical surgery), cirrhosis, polyarthritis and knee arthrosis	Hypertension, rheumatoid arthritis, osteoporosis and hip fracture	Diabetes type II, hypertension, diverticulosis and fever of unknown origin	Periodic depression, prostate cancer, cataract and dilation of aorta ascendens
Other medication	Promethazine, levothyroxine, dapagliflozin, metformin, atorvastatin, ezetimibe, clopidogrel and enalapril	Colchicine, spironolactone, pramipexole and clopidogrel	Methotrexate, bisphosphonates, prednisolone and clopidogrel	Enalapril, insulin glargine, atorvastatin, prednisolone, semaglutide, ezetimibe, dapagliflozin, clopidogrel and metformin	Modafinil, citalopram, zolpidem and lithium citrate

### PET imaging

PET imaging was performed as a 10-min recording on a Discovery 710 or MI PET/CT (GE Healthcare, Milwaukee, USA) at Bispebjerg Hospital (*n* = 4) or Siemens Biograph Vision 600 PET/CT scanner (Siemens Healthcare, Erlangen, Germany) at Aarhus University Hospital (*n* = 1, Modafinil case) 30 min after injection of 148 MBq [^18^F]FE-PE2I (range 122–162 MBq) or 200 MBq as previously described [5]. A low-dose CT was obtained for attenuation correction. Data were reconstructed into 3D datasets using a of a block-sequential regularized expectation maximization algorithm with a regularization parameter “β” of 250 (“Q.Clear”) or an iterative OSEM algorithm (8 iterations, 5 subsets) with resolution recovery (TrueX). The two scans for each patient were coregistered and manual volumes of interest were drawn in the caudate nucleus of the side with the highest uptake and in bilateral cerebellar grey matter including vermis using MIM Software (version 7.1, MIM Software Inc. Cleveland, OH, USA). The specific binding ratio (SBR) relative to non-specific binding of tracer in the reference region cerebellum that is devoid of DaT [12] was estimated as:

$$\mathrm{SBR}=\frac{{\mathrm{Activity}}_{\mathrm{Caudate}}-{\mathrm{Activity}}_{\mathrm{Cerebellum}}}{{\mathrm{Activity}}_{\mathrm{Cerebellum}}}$$

### Estimation of P-sertraline

We roughly estimated the sertraline plasma concentration (P-sertraline) at the time of the first scan using formula [13]:

$$\overline{C} = \frac{F \cdot D}{{{\text{Cl}} \cdot \tau }}$$

where $\overline{C}$ equals the average concentration at steady state, *F* equals the bioavailability of sertraline, which was set to 0.44 [14], *D* equals the dosage, Cl equals the clearance of sertraline, and *τ* equals the dosing interval. The clearance was determined using the following formula:

$${\text{Cl}} = k_{{\text{e}}} \cdot V_{{\text{d}}}$$

where *k*_e_ equals the elimination rate constant of sertraline, which was set to 0.019 h^−1^ [11] and *V*_d_ equals the volume of distribution, which was estimated by multiplying the patient’s weight by 75 mL kg^−1^ or 65 mL kg^−1^ for men and women, respectively. P-sertraline at time of the second scan (5-day sertraline pause) was assumed to equal zero.

### Statistics

The statistical significance was set to an alpha-level of *p* < 0.05. A paired t test was used to compare SBR with and without sertraline. A linear regression of the absolute change in SBR to the estimated change in P-sertraline was performed for each patient and the slope coefficients were averaged to achieve a common change in SBR as a function of P-sertraline.

## Results

**Fig. 1:**
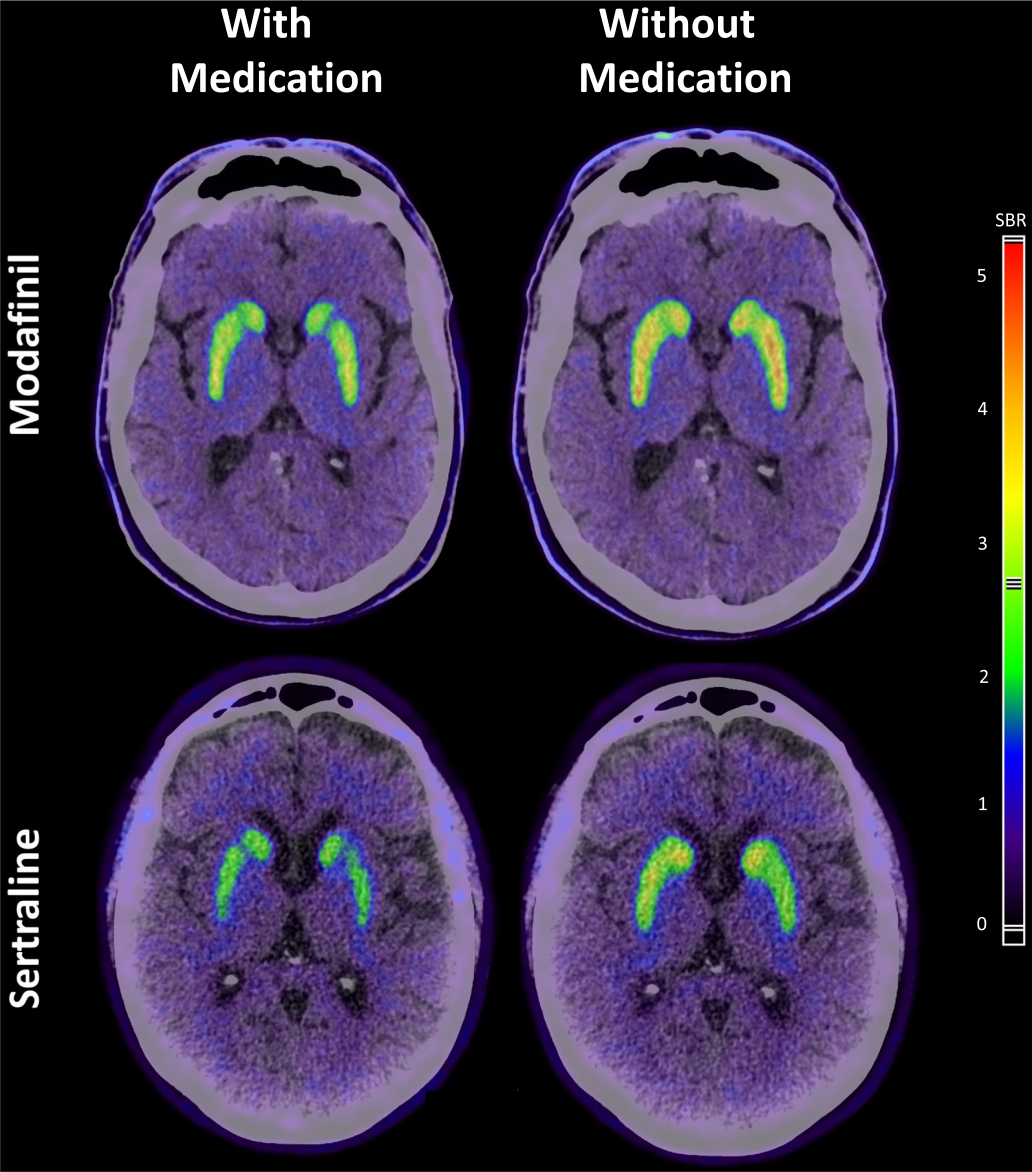
Axial sections of fused [^18^F]FE-PE2I PET and CT for visual comparison with and without 100-mg Modafinil (top) or 100-mg sertraline treatment (bottom) (patient 2)

**Fig. 2:**
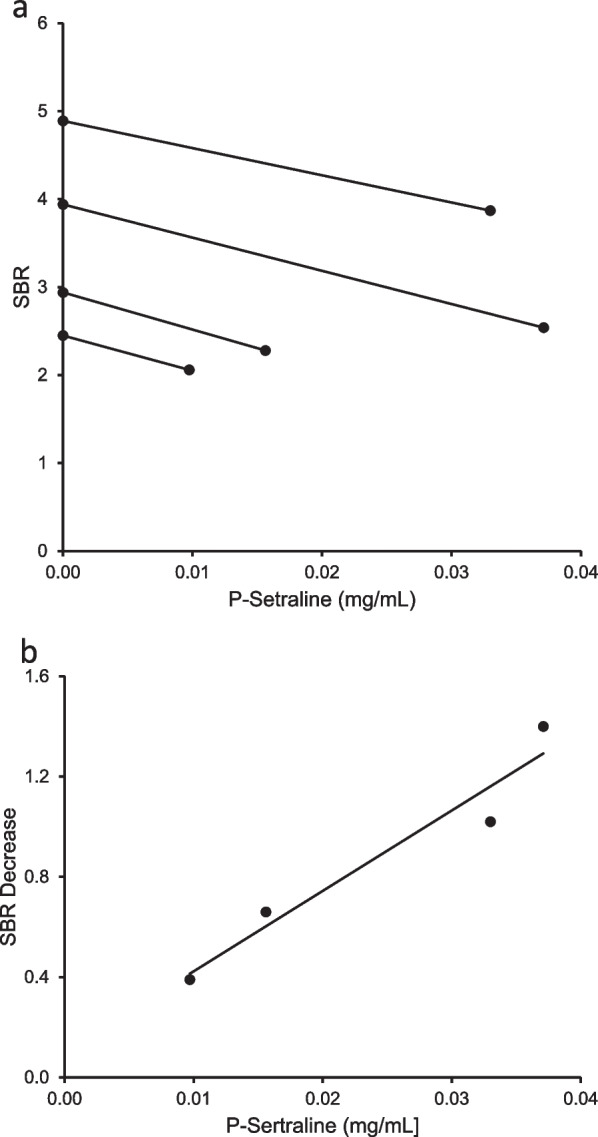
**a** SBR as a function of estimated P-sertraline at time of scan. The mean of slope coefficients was calculated to equal − 38 mL/mg. **b** The absolute reduction of SBR as a function of P-sertraline. The line of regression is shown. The sertraline pausing increases in SBR as a function of the estimated P-sertraline before sertraline pause

Visual analysis indicated a globally lower DaT activity when taking sertraline compared to the scans after 5-day sertraline pause (Fig. 1, Additional file 1: Fig. S1). SBR of caudate nucleus with and without sertraline showed a significant effect of pausing sertraline (*p* = 0.03) and the changes in SBR were similar to the change induced by 100 mg Modafinil (Table 2). We found no change in right-left asymmetry or the ratio between nucleus caudatus and putamen, i.e. the distribution pattern was unaltered. The mean of slope coefficients was calculated to equal − 38 mL/mg, i.e. SBR is reduced by 0.38 for each increase in P-sertraline of 0.01 mg/mL (Fig. 2a). SBR at the two scans as a function of the estimated P-sertraline showed a linear dose-dependent effect (Fig. 2b).

**Table 2:** SBR values of the caudate nucleus in the hemisphere with highest uptake with and without sertraline for all four patients

	With medication (SBR)	Without medication (SBR)	Change (absolute)	Change (relative) (%)
Patient 1	2.06	2.45	0.39	16
Patient 2	2.54	3.94	1.40	36
Patient 3	3.87	4.89	1.02	21
Patient 4	2.28	2.94	0.66	22
Modafinil	3.11	4.38	1.27	29

For comparison, we have included SBR-values for a patient with and without 100 mg Modafinil daily, a drug known to reduce DaT-activity and lead to false positive findings

## Discussion

In four patients suspected of Parkinson’s disease, we found a sertraline-induced significant dose-dependent reduction of [^18^F]FE-PE2I SBR in caudate nucleus that has not previously been described. The findings are in line with sertraline’s known high binding affinity for the DaT and may possess clinical implications due to the rather frequent use of sertraline. Thus, possible sertraline medication must be kept in mind when interpreting [^18^F]FE-PE2I PET scans. Other SSRIs have much lower affinity for DaT and should not influence DaT imaging. It is not known to what degree these findings are transferable to other radiolabelled DaT tracers, as [^123^I]FP-CIT show increased SBR during SSRI treatment [15]. Thus, we do not know how [^123^I]FP-CIT SPECT is influenced by sertraline. PET tracers targeting the dopamine synthesis, e.g. [^18^F]FDOPA or vesicular monoamine transporter, [^18^F]FP-DTBZ, should not be susceptible to sertraline. Optimally, sertraline should be discontinued before [^18^F]FE-PE2I PET, but the risk of a discontinuation syndrome with flu-like symptoms, imbalance, strange skin sensations, hyperarousal, and insomnia needs to be kept in mind [16, 17].

The SBR reduction induced by sertraline is in the order of magnitude of daily treatment with 100 mg Modafinil (normal dose for elderly patients), which is known to reduce DaT-activity.

The magnitude of [^18^F]FE-PE2I reduction significantly influencing the interpretation of DaT imaging may be highly variable between patients as normal limits of striatal SBR are from 3 to 7 on our scanner system. We found that SBR is reduced by 0.38 for each increase in P-sertraline of 0.01 mg/mL. Thus, a 75-kg male taking 50 mg per day results in a SBR reduction of 0.32, while a 65-kg female is reduced by 0.44. Depending on bodyweight and gender, a dose of 50 mg may be acceptable if the reduction is kept in mind when interpreting the scan. Higher doses can be switched to another SSRI with low affinity for the DaT such as escitalopram [18]. Alternatively, only patients with equivocal scan results could be rescanned after a 5-day sertraline pause as most patients with neurodegenerative diseases affecting the dopamine system show a characteristic “neurodegenerative pattern”. Such patterns including reduced binding in the posterior putamen or sometimes striking left–right asymmetry are not expected with pharmacological DaT blockade, where the entire striatum shows more globally and homogeneously reduced binding.

There are limitations to the study, including the sample size. However, the dose-dependent decrease in all subjects combined with sertraline’s high affinity for DaT supports the influence of sertraline for [^18^F]FE-PE2I PET imaging. Further, [^18^F]FE-PE2I PET has previously been shown to have good test–retest reproducibility [19, 20] supporting that the present results are trustworthy. All patients had a number of comorbidities, which may have influenced the results. Further, only patients with equivocal scan results were rescanned as the study was performed in a clinical setting. And a number of patients who despite of sertraline usage still showed marked asymmetry due to Parkinson’s disease may not even have been identified.

The biological half-life of sertraline is highly variable and discontinuation for five days may not be sufficient in all cases, and remaining sertraline in plasma or receptor bound may still be interfering with the second [^18^F]FE-PE2I PET. Furthermore, the major metabolite of sertraline, *N-*desmethyl-sertraline, has a half-life of 60–70 h and a low *K*_D_ for DaT at 129 nM [9, 14]. Thus, our findings may have underestimated the blocking effect of sertraline. The present study cannot robustly determine a cut-off value for daily sertraline ingestion or determine the optimal length for discontinuation. The suggested cut-off of 50 mg and 5-day sertraline pause is a rough estimate, and a prospective dose response study is warranted.

In conclusion, the significant dose-dependent reduction of [^18^F]FE-PE2I uptake in sertraline-treated patients should be taken into account in future [^18^F]FE-PE2I PET imaging.

## Supplementary Information

**Additional file 1. Fig S1:** Axial sections of fused [^18^F]FE-PE2I PET and CT for visual comparison with and without sertraline for all four patients in sertraline treatment.

## Data Availability

All data generated or analysed during this study are included in anonymous form in this published article.
