# Supplementary material for: Sertraline treatment influences [18F]FE-PE2I PET imaging for Parkinsonism
Source: EJNMMI Res. 2023 May 23;13:46. doi: 10.1186/s13550-023-01000-6 (PMC10205964; doi:10.1186/s13550-023-01000-6)
Supplement: Supplementary file 1 — Additional file 1. Fig S1: Axial sections of fused [18F]FE-PE2I PET and CT for visual comparison with and without sertraline for all four patients in sertraline treatment. [file 13550_2023_1000_MOESM1_ESM.docx]

#
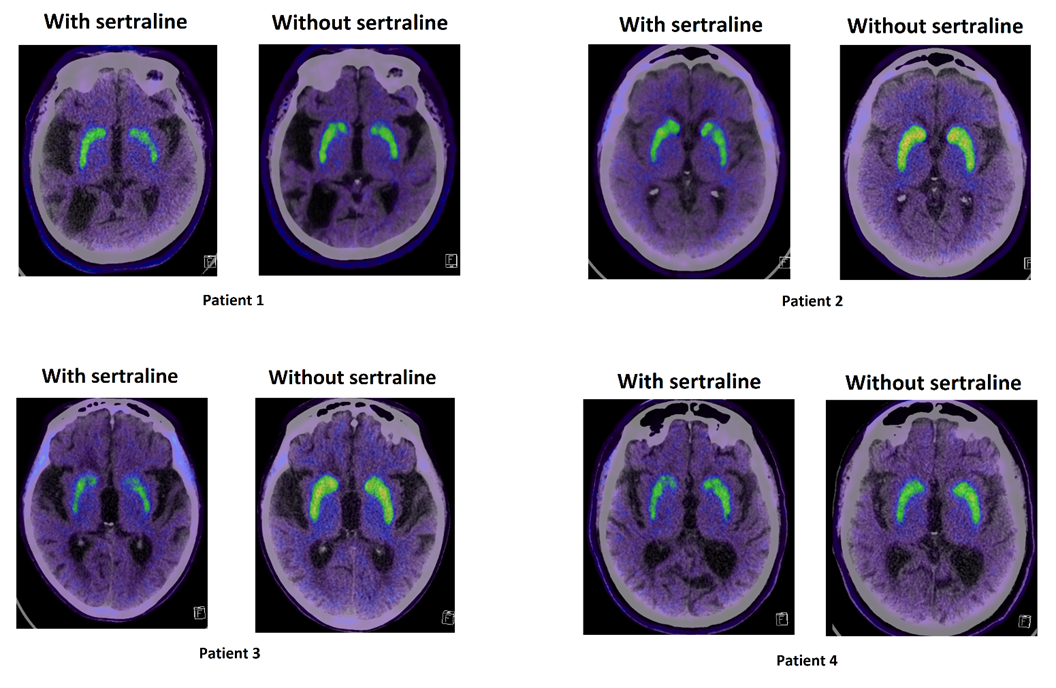
Supplementary material

Supplementary Figure 1: Axial sections of fused [^18^F]FE-PE2I PET and CT for visual comparison with and without sertraline for all four patients in sertraline treatment.
